# GRE and Undergraduate GPA as Predictors of Veterinary Medical School Grade Point Average, VEA Scores and NAVLE Scores While Accounting for Range Restriction

**DOI:** 10.3389/fvets.2020.576354

**Published:** 2020-10-28

**Authors:** Jared A. Danielson, Rebecca G. Burzette

**Affiliations:** College of Veterinary Medicine, Iowa State University, Ames, IA, United States

**Keywords:** academic achievement, admission predictors, GPA, GRE verbal, veterinary medical education, restriction of range, Veterinary Educational Assessment (VEA), North American Veterinary Licensing Exam (NAVLE)

## Abstract

We explored the relation between Undergraduate GPA (UGPA) and Graduate Record Examination (GRE) Verbal scores and several indices of achievement in veterinary medical education across five cohorts of veterinary students (N per model ranging from 109 to 143). Achievement indices included overall grade point average in veterinary school (CVMGPA), scores on the North American Veterinary Licensing Examination (NAVLE) and scores on the Veterinary Educational Assessment (VEA). We calculated zero order correlations among all measures, and corrected correlations for range restriction. In all cases, corrected correlations exceeded uncorrected ones. For each index of achievement, we conducted hierarchical regressions using the corrected correlations as input, entering UGPA in the first step and GRE Verbal in the second step. Overall, UGPA and GRE Verbal combined explained from 70 to 84% of variance in CVMGPA, 51–91% of variance in VEA scores, and 41–92% of variance in NAVLE scores. For 12 of 15 comparisons, the second step (including GRE Verbal scores) significantly improved *R*^2^. Our results reaffirm the value of UGPA scores and GRE Verbal scores for predicting subsequent academic achievement in veterinary school.

## Introduction

Graduate and professional programs seek to admit applicants who will succeed academically and in subsequent careers. Additionally, programs often utilize admissions policies to meet other objectives, such as recognizing important non-academic indicators of success or merit, and ensuring fairness for all applicants.

One common predictor of success in veterinary medical education programs is undergraduate grade point average (UGPA). Research supports UGPA's utility in predicting achievement in veterinary school (1–10).

The Graduate Record Examination (GRE), a standardized examination offered by Educational Testing Services, is intended to predict achievement for graduate and professional school, and, like undergraduate GPA, is commonly used for admissions decisions in veterinary medical education. Researchers have explored the GRE's utility for predicting subsequent achievement for a wide range of disciplines. In a meta-analysis synthesizing research from 1,521 students, Kuncel et al. (11) found the GRE to have significant power to predict achievement in graduate programs, and Kuncel et al. (12) found that significance to be present for both doctoral and master's level study across a wide range of disciplines.

Veterinary programs have long relied upon the Graduate Record Examination (GRE) for informing admissions decisions, a practice that is supported in the literature, with decades of studies showing a positive relation between GRE scores or sub-scores and subsequent achievement (1–7, 13–15).

Some studies, however, have cast some doubt on the efficacy of the GRE, finding that the GRE provided little or no added predictive value, when combined with other common predictors such as grade point average in programs such as nurse anesthesiology (16), biomedical PhD programs (17, 18); physician assistant programs (19); nursing (20), construction management (21) and developmental biology (22). Additionally, the GRE has been criticized because it has been shown in some cases to under-predict success for women (23), and to present a barrier to application for otherwise qualified applicants (overall) (24) as well as underrepresented applicants (25).

Defenders of the GRE point to its overall track record (26), and note that representation of historically disadvantaged groups in graduate study has increased even as the GRE has been utilized for admissions decisions (27). Furthermore, many studies underestimate its predictive power by relying on simple univariate correlative approaches that do not account for factors such as restriction of range (3, 26). Additionally, standardized tests, generally, might play an important role in making the admissions process fairer for applicants. For instance, Schwager et al. (28) found the GRE to be an important admissions tool because they were recruiting students from an international population, and the countries supplying their applicants differed systematically in grading practices, thereby making it difficult to compare applicants fairly using GPA alone. They found that GRE scores were useful in predicting graduate GPA above and beyond undergraduate GPA, independent of students' socioeconomic status.

Despite the available literature, therefore, more studies are needed that investigate the characteristics and usefulness of standardized tests such as the GRE, providing data to contribute to meta-analysis, and overcoming the shortcomings of many existing studies that failed to account for restriction of range. The present study sought to address this need by answering the following questions for a large veterinary college in the Midwestern United States:

1. Accounting for restriction of range, what is the predictive relation between GRE scores and indicators of achievement in veterinary medicine, including veterinary grade point average (CVMGPA), score on the Veterinary Educational Assessment (VEA), and score on the North American Veterinary Licensing Exam (NAVLE)?
2. Do GRE scores provide predictive power beyond undergraduate GPA (UGPA)?

## Materials and Methods

This study was reviewed and declared exempt by the Iowa State University Institutional Review Board, #19-243-00.

### Participants

We obtained data from the admissions office of one veterinary medicine college in the Midwestern United States. Participants included 2013, 2014, 2015, 2016, and 2017 graduates who had complete data for undergraduate GPA, GRE scores, and three indices of subsequent achievement, including Veterinary Medicine Cumulative GPA (CVMGPA), Veterinary Educational Assessment (VEA) scores, and North American Veterinary Licensing Exam (NAVLE) scores. Table 1 presents the Ns, percent male, and mean age of the students at graduation, by cohort and outcome.

**Table 1:** Demographics (gender and age) by cohort and outcome.

**Cohort**	**VEA**				**NAVLE**				**CVMGPA**			
	***N***	**% Male**	**Mean age**	**SD age**	***N***	**% Male**	**Mean age**	**SD age**	***N***	**% Male**	**Mean age**	**SD age**
2013	136	35%	27.7	2.93	121	36%	27.2	2.03	139	35%	27.7	2.92
2014	143	29%	27.9	3.77	113	30%	28.0	4.11	143	29%	27.9	3.77
2015	122	25%	27.3	2.76	109	25%	27.3	2.88	122	25%	27.3	2.76
2016	131	25%	27.5	2.24	121	26%	27.5	2.27	132	25%	27.5	2.26
2017	138	24%	27.7	2.88	122	24%	27.6	2.66	138	24%	27.7	2.88

### Measures

#### Undergraduate GPA

Undergraduate GPA (UGPA) is the final cumulative GPA from the student's undergraduate years. For the purpose of adjusting for restriction of range, the mean and standard deviation for UGPA in the unrestricted sample were obtained from a published article (29), and based on data from 62,122 students at 26 institutions between 2000 and 2005.

#### GRE Scores

We conducted a series of regression analyses using observed correlations to predict our three indices of subsequent achievement (VEA, NAVLE, and Veterinary Medicine Cumulative GPA) using UGPA and three available GRE sub scores (Verbal, Quantitative, and Writing). Results showed that GRE Verbal was the only GRE score that significantly predicted subsequent achievement (See Table 2). Therefore, for subsequent analyses, we used only the Verbal Reasoning subtest of the GRE. The mean and standard deviation for GRE Verbal for the unrestricted sample were obtained from the Educational Testing Services website (www.ets.org/s/gre/pdf/gre_guide.pdf) and were based on data from >1 million test-takers collected between 2016 and 2018.

**Table 2:** Regressions with observed correlations - GRE sub-scores and dependent variables.

**Year**	**2013**	**2014**	**2015**	**2016**	**2017**
**Predicting CVMGPA**					
*N*	139	143	122	132	138
Adj. *R^2^*	0.249	0.242	0.250	0.216	0.268
UG GPA β	0.400^***^	0.460^***^	0.485^***^	0.473^***^	0.489^***^
GRE verbal β	0.190^*^	0.171^*^	0.055	0.021	0.207^*^
GRE quant β	0.087	−0.033	0.119	0.083	0.009
GRE write β	0.092	0.044	0.029	0.013	0.004
**Predicting NAVLE**					
*N*	121	113	109	121	122
Adj. *R^2^*	0.103	0.223	0.222	0.124	0.221
UG GPA β	0.194^*^	0.362^***^	0.316^***^	0.300^**^	0.356^***^
GRE verbal β	0.260^**^	0.344^***^	0.329^**^	0.262^**^	0.335^**^
GRE quant β	0.097	−0.132	0.105	−0.029	−0.019
GRE write β	−0.018	−0.028	−0.137	−0.047	0.016
**Predicting VEA**					
*N*	136	143	122	131	138
Adj. *R^2^*	0.216	0.239	0.195	0.124	0.257
UG GPA β	0.306^***^	0.300^***^	0.304^***^	0.217^**^	0.318^***^
GRE verbal β	0.348^***^	0.394^***^	0.330^***^	0.278^**^	0.384^***^
GRE quant β	0.065	−0.026	0.074	0.069	0.060
GRE write β	−0.037	−0.046	−0.038	−0.050	−0.019

*** *p < 0.001*.

** *p < 0.01*.

* *p < 0.05*.

#### Three Indices of Subsequent Achievement

The three indices of subsequent achievement included Veterinary Educational Assessment (VEA) scores, North American Veterinary License Exam (NAVLE) scores, and Veterinary Medicine Cumulative GPA (CVMGPA).

The VEA is a 300-item, multiple-choice pre-clinical examination that was designed to assess basic proficiency in the areas of veterinary anatomy, physiology, pharmacology, microbiology, and pathology, developed by the International Council for Veterinary Assessment (ICVA) and the National Board of Veterinary Medical Examiners. The VEA is developed external to the College of Veterinary Medicine where the study was conducted, and is purchased by the College for the purpose of benchmarking student achievement in the basic sciences.

The NAVLE scores are students' scores on the North American Veterinary Licensing Examination. The NAVLE is an electronically administered, 360-item, multiple-choice examination that is required for veterinary licensure in the United States and Canada. The NAVLE is developed and administered external to the college, and students were not required to release their NAVLE scores to the college.

Veterinary Medicine GPA (CVMGPA) is students' cumulative grade point average at the end of their 4 years in the veterinary college.

As seen in Table 1, Ns vary somewhat across measures, with NAVLE data generally being available for fewer numbers of participants than VEA or CVMGPA data. In 2013 and 2016 there were also slightly fewer VEA scores available than CVMGPA values (three in 2013 and one in 2016). Some NAVLE data were unavailable for each cohort because releasing NAVLE data to the college was voluntary, and not all students chose to release it. Four VEA scores were unavailable because those students were unable to take the exam due to illness.

### Method

We conducted a series of correlations among UGPA, GRE Verbal Reasoning, and the three indices of achievement. Then, because these correlations were range restricted, we corrected the correlations following Wiberg and Sundström (30), using a formula that has been “widely discussed and used (31–35). The formula uses the correlation of the restricted sample and the standard deviation of the independent variable (X) in the restricted sample and in the unrestricted sample to provide an estimate of the correlation in the population (p. 4).” Table 3 contains a comparison of standard deviations for both UGPA and GRE verbal between the study cohorts and the wider population.

**Table 3:** Comparison of standard deviations for the study cohort and wider population.

**Predictor**	**Outcome**	**Range of sample standard deviations**	**Population standard deviation**
UGPA	CVMGPA	0.241–0.286	0.79
UGPA	NAVLE	0.240–0.276	0.79
UGPA	VEA	0.243–0.337	0.79
GREVERBAL	CVMGPA	5.094–5.270	8.43
GREVERBAL	NAVLE	5.117–5.290	8.43
GREVERBAL	VEA	5.094–5.291	8.43

Finally, we conducted hierarchical regressions using the corrected correlations as input, entering UGPA in the first step and GRE Verbal in the second step. The NAVLE, VEA, and GPA would be expected to produce similar results across years with similar cohorts. In the present study, cohorts are similar, in that they were recruited to the same college of veterinary medicine using nearly identical admissions processes. Nonetheless, there are modest but systematic differences among cohorts. Because such differences could affect the results, we sought to eliminate the possibility that an exceptionally strong (or weak) relation within one or more cohorts might misleadingly distort the relation for another cohort. Therefore, we conducted these analyses separately for each cohort and each outcome.

## Results

### VEA Scores

Table 4 contains the observed and corrected correlations, as well as the means and standard deviations for predicting VEA scores for each of the five cohorts. Table 5 contains the hierarchical regression results by cohort, using corrected correlations. Step 1 of the hierarchical regression contained UGPA. Step 2 included UGPA and GRE Verbal. With the exception of the class of 2014, the second step in the models significantly improved *R*^2^.

**Table 4:** Observed and corrected correlations predicting VEA for classes of 2013–2017.

	**Class of 2013 (*N* = 136)**			**Class of 2014 (*N* = 143)**			**Class of 2015 (*N* = 122)**			**Class of 2016 (*N* = 131)**			**Class of 2017 (*N* = 138)**		
	**UGPA**	**GRE verbal**	**VEA**	**UGPA**	**GRE verbal**	**VEA**	**UGPA**	**GRE verbal**	**VEA**	**UGPA**	**GRE verbal**	**VEA**	**UGPA**	**GRE verbal**	**VEA**
UGPA	1.00	0.07	0.34	1.00	0.13	0.34	1.00	−0.05	0.30	1.00	0.07	0.24	1.00	0.02	0.33
GRE verbal	0.20	1.00	0.37	0.35	1.00	0.42	−0.15	1.00	0.34	0.22	1.00	0.31	0.06	1.00	0.41
VEA	0.71	0.54	1.00	0.72	0.61	1.00	0.72	0.50	1.00	0.63	0.46	1.00	0.72	0.59	1.00
Mean^a^	3.17	150.05	61.24	3.17	150.05	65.76	3.17	150.05	58.60	3.17	150.05	64.43	3.17	150.05	63.83
SD^b^	0.79	8.43	8.40	0.79	8.43	9.34	0.79	8.43	8.85	0.79	8.43	8.96	0.79	8.43	8.30

*Observed correlations are above the diagonal; corrected correlations are below the diagonal*.

a *Means for UGPA and GRE Verbal are estimated population means; mean for VEA is sample mean*.

b *Standard deviations for UGPA and GRE Verbal are estimated population SDs; SD for VEA is sample SD*.

**Table 5:** Hierarchical regressions predicting VEA, by cohort.

**Cohort**	***B***	***SE B***	**β**	***R^**2**^***	**Adj. *R^**2**^***	**Δ *R^**2**^***	**F Δ *R^**2**^***
2013							
Step 1				0.504	0.500	0.504	136.216^***^
UGPA	21.421	1.835	0.710^***^				
Step 2				0.669	0.664	0.165	66.322^***^
UGPA	18.920	1.536	0.627^***^				
GRE verbal	0.658	0.081	0.415^***^				
2014							
Step 1				0.518	0.515	0.518	151.774^***^
UGPA	24.688	2.004	0.720^***^				
Step 2				0.664	0.660	0.146	60.939
UGPA	19.792	1.792	0.577^***^				
GRE verbal	0.748	0.096	0.408^***^				
2015							
Step 1				0.518	0.514	0.518	130.246^***^
UGPA	26.053	2.283	0.720^***^				
Step 2				0.909	0.908	0.391	515.881^***^
UGPA	29.845	1.007	0.815^***^				
GRE verbal	1.074	0.047	0.632^***^				
2016							
Step 1				0.397	0.392	0.397	84.895^***^
UGPA	23.300	2.529	0.630^***^				
Step 2				0.512	0.505	0.115	30.291^***^
UGPA	20.467	2.340	0.553^***^				
GRE verbal	0.596	0.108	0.348^***^				
2017							
Step 1				0.518	0.515	0.518	146.392^***^
UGPA	22.075	1.825	0.720^***^				
Step 2				0.818	0.816	0.300	223.157^***^
UGPA	21.066	1.126	0.687^***^				
GRE verbal	0.871	0.058	0.549^***^				

*** *p < 0.001*.

Across the cohorts, UGPA accounted for from 39 to 52% of the variance in VEA scores. When GRE Verbal was added, the models accounted for from 51 to 91% of the variance in VEA scores.

### NAVLE Scores

Table 6 contains the observed and corrected correlations, as well as the means and standard deviations for predicting NAVLE scores for each of the five cohorts. Table 7 contains the hierarchical regression results by cohort, using corrected correlations. Step 1 of the hierarchical regression contained UGPA. Step 2 included UGPA and GRE Verbal. The second step in the models significantly improved *R*^2^ for all cohorts. Across the cohorts, UGPA accounted for from 29 to 57% of the variance in NAVLE scores. When GRE Verbal was added, the models accounted for from 41 to 92% of the variance in NAVLE scores.

**Table 6:** Observed and corrected correlations predicting NAVLE for classes of 2013–2017.

	**Class of 2013 (*N* = 121)**			**Class of 2014 (*N* = 113)**			**Class of 2015 (*N* = 109)**			**Class of 2016 (*N* = 121)**			**Class of 2017 (*N* = 122)**		
	**UGPA**	**GRE verbal**	**NAV LE**	**UGPA**	**GRE verbal**	**NAV LE**	**UGPA**	**GRE verbal**	**NAV LE**	**UGPA**	**GRE verbal**	**NAV LE**	**UGPA**	**GRE verbal**	**NAV LE**
UGPA	1.00	0.05	0.22	1.00	0.09	0.35	1.00	−0.02	0.33	1.00	0.04	0.31	1.00	0.05	0.37
GRE verbal	0.15	1.00	0.29	0.27	1.00	0.37	−0.08	1.00	0.36	0.13	1.00	0.25	0.15	1.00	0.35
NAV LE	0.54	0.44	1.00	0.76	0.54	1.00	0.74	0.54	1.00	0.73	0.39	1.00	0.76	0.51	1.00
Mean^a^	3.17	150.05	508.90	3.17	150.05	512.49	3.17	150.05	503.3	3.17	150.05	496.28	3.17	150.05	507.52
SD^b^	0.79	8.43	59.04	0.79	8.43	62.01	0.79	8.43	52.13	0.79	8.43	61.65	0.79	8.43	53.53

*Observed correlations are above the diagonal; corrected correlations are below the diagonal*.

a *Means for UGPA and GRE Verbal are estimated population means; mean for NAVLE is sample mean*.

b *Standard deviations for UGPA and GRE Verbal are estimated population SDs; SD for NAVLE is sample SD*.

**Table 7:** Hierarchical regressions predicting NAVLE, by cohort.

**Cohort**	***B***	***SE B***	**β**	***R^**2**^***	**Adj. *R^**2**^***	**Δ *R^**2**^***	**F Δ *R^**2**^***
2013							
Step 1				0.292	0.286	0.292	48.984^***^
UGPA	115.675	16.528	0.540^***^				
Step 2				0.423	0.414	0.132	26.985^***^
UGPA	103.874	15.145	0.485^***^				
GRE verbal	4.207	0.810	0.367^***^				
2014							
Step 1				0.578	0.574	0.578	151.784^***^
UGPA	189.108	15.350	0.760^***^				
Step 2				0.699	0.693	0.121	44.112^***^
UGPA	164.846	13.529	0.662^***^				
GRE verbal	4.333	0.652	361^***^				
2015							
Step 1				0.563	0.558	0.563	138.857^***^
UGPA	159.309	13.519	0.750^***^				
Step 2				0.925	0.923	0.362	515.663^***^
UGPA	169.571	5.649	0.798^***^				
GRE verbal	6.135	0.270	0.604^***^				
2016							
Step 1				0.533	0.529	0.533	135.763^***^
UGPA	187.719	16.111	0.730^***^				
Step 2				0.621	0.615	0.089	27.614^***^
UGPA	177.684	14.689	0.691^***^				
GRE verbal	3.598	0.685	0.300^***^				
2017							
Step 1				0.578	0.574	0.578	164.091^***^
UGPA	157.359	11.894	0.760^***^				
Step 2				0.738	0.734	0.160	72.872^***^
UGPA	140.176	9.514	0.699^***^				
GRE verbal	4.099	0.480	0.405^***^				

*** *p < 0.001*.

### CVMGPA

Table 8 contains the observed and corrected correlations, as well as the means and standard deviations for predicting CVMGPA scores for each of the five cohorts. Table 9 contains the hierarchical regression results by cohort, using corrected correlations. Step 1 of the hierarchical regression contained UGPA. Step 2 included UGPA and GRE Verbal. With the exception of the class of 2014 and the class of 2016, the second step in the models significantly improved *R*^2^ for the cohorts. Across the cohorts, UGPA accounted for from 65 to 77% of the variance in CVMGPA scores. When GRE Verbal was added, the models accounted for from 70 to 84% of the variance in CVMGPA scores.

**Table 8:** Observed and corrected correlations predicting CVMGPA for classes of 2013–2017.

	**Class of 2013 (*N* = 139)**			**Class of 2014 (*N* = 143)**			**Class of 2015 (*N* = 122)**			**Class of 2016 (*N* = 132)**			**Class of 2017 (*N* = 138)**		
	**UGPA**	**GRE verbal**	**CVM GPA**	**UGPA**	**GRE verbal**	**CVM GPA**	**UGPA**	**GRE verbal**	**CVM GPA**	**UGPA**	**GRE verbal**	**CVM GPA**	**UGPA**	**GRE verbal**	**CVM GPA**
UGPA	1.00	0.10	0.45	1.00	0.13	0.48	1.00	−0.05	0.50	1.00	0.07	0.48	1.00	0.02	0.50
GRE verbal	0.26	1.00	0.28	0.35	1.00	0.23	−0.15	1.00	0.08	0.23	1.00	0.09	0.06	1.00	0.22
CVM GPA	0.81	0.42	1.00	0.85	0.37	1.00	0.88	0.13	1.00	0.87	0.15	1.00	0.86	0.35	1.00
Mean^a^	3.17	150.05	3.25	3.17	150.05	3.25	3.17	150.05	3.24	3.17	150.05	3.31	3.17	150.05	3.26
SD^b^	0.79	8.43	0.33	0.79	8.43	0.322	0.79	8.43	0.304	0.79	8.43	0.333	0.79	8.43	0.300

*Observed correlations are above the diagonal; corrected correlations are below the diagonal*.

a *Means for UGPA and GRE Verbal are estimated population means; mean for CVMGPA is sample mean*.

b *Standard deviations for UGPA and GRE Verbal are estimated population SDs; SD for CVMGPA is sample SD*.

**Table 9:** Hierarchical regressions predicting CVMGPA, by cohort.

**Cohort**	***B***	***SE B***	**β**	***R^**2**^***	**Adj. *R^**2**^***	**Δ *R^**2**^***	**F Δ *R^**2**^***
2013							
Step 1				0.656	0.654	0.656	261.372^***^
UGPA	0.945	0.058	0.810^***^				
Step 2				0.703	0.699	0.047	21.544^***^
UGPA	0.877	0.056	0.752^***^				
GRE verbal	0.014	0.003	0.225^***^				
2014							
Step 1				0.722	0.721	0.722	367.108^***^
UGPA	1.005	0.052	0.850^***^				
Step 2				0.728	0.725	0.006	3.089
UGPA	0.971	0.056	0.821^***^				
GRE verbal	0.005	0.003	0.083				
2015							
Step 1				0.774	0.773	0.774	415.348^***^
UGPA	1.096	0.054	0.880^***^				
Step 2				0.839	0.837	0.065	48.532^***^
UGPA	1.144	0.046	0.919^***^				
GRE verbal	0.015	0.002	0.258^*^				
2016							
Step 1				0.757	0.755	0.757	404.759^***^
UGPA	1.198	0.060	0.870				
Step 2				0.760	0.756	0.003	1.422
UGPA	1.215	0.061	0.882^***^				
GRE verbal	−0.003	0.003	−0.053				
2017							
Step 1				0740	0.738	0.740	386.273^***^
UGPA	0.953	0.048	0.860^***^				
Step 2				0.829	0.826	0.089	70.536^***^
UGPA	0.933	0.040	0.842^***^				
GRE verbal	0.017	0.002	0.299^***^				

* *p < 0.05*,

*** *p < 0.001*.

## Discussion

Overall, both UGPA and GRE-Verbal contributed significantly to explaining achievement on the outcome variables. As seen in Tables 4, 6, 8, the overall size of the effects from UGPA tended to be larger than the effects from GRE-Verbal. Furthermore, because UGPA tends to precede GRE scores, and because it is generally more ubiquitously available than GRE scores, we entered UGPA into each regression model first. As a result of these two factors, UGPA consistently explained more variance in each model than did GRE-Verbal scores.

### Veterinary Educational Assessment

We found no research examining the VEA. However, two studies examined the Qualifying Exam (QE), an earlier version of the VEA. Danielson et al. (4) found that the Qualifying Exam (QE) was a significant predictor of success on the NAVLE Examination, and a mediator for the effect of UGPA and GRE (combined) scores for explaining success on the NAVLE. Similarly, Fuentalba et al. (5) found that GRE Quantitative scores were good predictors of QE scores, and found that QE scores correlated with GPA in all years of the curriculum.

Like the QE, the VEA was designed to measure veterinary students' mastery of the basic science knowledge undergirding diagnostic and clinical reasoning. Undergraduate GPA, which reflects how well-students perform in undergraduate courses prior to enrolling in veterinary school, could be associated with VEA scores in at least two ways. First, many of the undergraduate courses included in the UGPA calculation included content that was similar or prerequisite to content included in early courses in the veterinary curriculum. For instance, courses in General Chemistry, Organic Chemistry, Biochemistry, General Biology, Genetics, and Anatomy/Physiology contain knowledge undergirding subsequent learning of Veterinary Anatomy, Physiology, Pathology, Pharmacology, and Microbiology. Because VEA scores represent achievement in these subjects, it is reasonable to expect UGPA to predict VEA scores. Second, prerequisite undergraduate courses are focused on mastery of basic science rather than applied or clinical science. In this way, also, UGPA is aligned with the VEA. Across cohorts, in the present study variance in UGPA explained anywhere from 40 to 52% of variance in VEA scores, explaining 50% or more of variance in four out of five cohorts. This suggests that, for predicting students' ability to master basic science knowledge, UGPA is a powerful predictor.

The GRE Verbal test is designed to “assesses… ability to analyze and evaluate written material and synthesize information obtained from it, analyze relationships among component parts of sentences and recognize relationships among words and concepts” (36). Assuming the GRE Verbal test performed as designed, this ability should predict VEA scores independently of content knowledge or content-specific ability to analyze, evaluate, and synthesize information in medical basic sciences. The VEA uses formats that require analysis and evaluation of written material and synthesis of information, such as presenting scenarios involving disease or injury to animals, and requiring the examinee to select the most likely cause, or anatomical structure most likely involved (37) Of course, this ability should also be highly correlated with undergraduate GPA, since analysis, evaluation, and synthesis of verbal information should be associated with students' overall ability to perform well on coursework and course related exams. However, the GRE Verbal was able to explain variance in VEA scores independent of UGPA. Across all cohorts, the GRE Verbal score contributed significantly and meaningfully to the regression models, explaining an additional 12–30% of variability in VEA score, beyond the variation explained by UGPA alone.

If the VEA is an accurate indicator of veterinary student mastery of key basic science knowledge, then UGPA and GRE Verbal together contributed powerfully to our ability in the present study to predict students' subsequent basic science knowledge, together explaining between 51 and 91% of VEA test scores. Only in 2016 were these combined measures able to account for <2/3^rds^ of the variability in VEA scores.

### NAVLE

The NAVLE is intended to measure veterinary graduates' preparation to practice veterinary medicine across 13 species and four competency domains including clinical practice, communication, professionalism/practice management/wellness, and preventive medicine/animal welfare (38).

Few studies have explored relations between common pre-admission variables and NAVLE scores. Roush et al. (15) found a strong positive relationship between NAVLE scores and veterinary school GPA and veterinary class rank. They found low, but significant correlations between the NAVLE and the Verbal GRE, Total GRE, and pre-admission science GPA. Similarly, Danielson et al. (4) found modest correlations that were only inconsistently significant between GRE and NAVLE scores as well as between UGPA and NAVLE scores. They also found that both GRE and UGPA were significant indirect predictors of NAVLE scores through QE scores and/or VGPA. Molgaard et al. (6) similarly found that both UGPA and GRE scores (all combined) contributed modestly, but significantly to explaining NAVLE scores. None of the above researchers corrected their correlations for restriction of range. Both Roush et al. and Danielson et al. hypothesized that NAVLE scores were more strongly correlated with veterinary school performance than pre-admission variables because NAVLE scores better reflected what students did in veterinary school than the knowledge and skills needed to perform well on pre-admission measures. However, the strong corrected correlations in the present study introduce an alternative hypothesis. Perhaps the relatively weaker correlations found between prerequisite measures and NAVLE scores in the prior studies were a result of restriction of range. While the present study does not allow us to confirm either hypothesis, we recommend it be explored further.

As noted earlier, across each cohort, some NAVLE data were missing because students were not required to release their NAVLE scores. This could have affected the results of the analysis, particularly if students who chose to withhold their scores differed in some systematic way from other students. For instance if students who withheld their NAVLE scores systematically performed differently on the other variables, compared to other students, than they did on the NAVLE, then the strength of the relations may be over-estimated. Similarly, if consistently high or low scoring students chose to withhold their NAVLE scores, the strength of the correlations might be somewhat underestimated. However, the fact that the majority of students shared their NAVLE scores, combined with the strong correlations among NAVLE scores and other scores, suggest that sufficient students performing across a wide range of scores chose to share their scores that the overall nature of the relations among variables was detectable.

### CVMGPA

Overall GPA during the veterinary school program should provide a broad indication of students' achievement in veterinary medicine across the basic and clinical sciences, and in both classroom and applied clinical contexts. As discussed earlier, long-standing research supports significant, though often modest positive relationships between undergraduate GPA and grades in veterinary school. This holds true for a variety of GPA calculations, including after Year 1 (2, 3, 7, 10), at various points throughout the program (1, 5, 6), and at the end of the program (4, 8, 9). Of those studies, only Powers' study corrected for restriction of range. The multiple correlations found by Powers for the effect of GRE (V, Q and A) and UGPA were, on average, 0.76, similar to the range of 0.70–0.84 found in the present study. This finding suggests that the more modest relations between CVMGPA and pre admissions GPA found in prior studies may have been influenced by restriction of range.

### GRE Sub-Scores

Prior studies have reported significant correlations between GRE sub-scores and achievement in veterinary school, with a variety of outcomes. Four studies (1–3, 5) found that the GRE quantitative and analytical scores were the best predictors of subsequent achievement. Confer and Lorenz (2) found that the GRE Biology sub score was also a good predictor of subsequent achievement. A number of researchers (4, 6, 7, 14) combined multiple GRE sub section scores, and in all cases the combined scores were significant predictors of subsequent achievement. Like the current study, two prior studies found the GRE Verbal score to be a better predictor of subsequent achievement than other sub-scores for some dependent variables. Kelman (13) found that the GRE Verbal sub-score contributed significantly to a regression model predicting cumulative veterinary college GPA, whereas the quantitative sub score did not. Roush et al. (15) found that only the GRE Verbal sub score was significantly correlated with NAVLE score, whereas only the GRE quantitative and analytical sub scores correlated significantly with veterinary school GPA. In the present study the GRE Verbal sub-score consistently correlated more powerfully with subsequent measures than either the GRE Quantitative or GRE Writing scores and, when combined in one regression analysis, was the only sub-score that contributed significantly to regression models. It is unclear why GRE sub-scores do not appear to contribute consistently across studies. The variety of research approaches and dependent variables in existing studies do not reveal unambiguous patterns. This question deserves more study.

### Implications

The present study highlights the value of correcting for range restriction when estimating the contribution of variables such as GPA and GRE to explaining subsequent achievement. Correcting for restriction of range, UGPA and GRE together explained as much as 84, 91, and 92% of the variance in CVMGPA, VEA, and NAVLE scores, respectively. There are at least two important implications of this finding. First, prior studies may have substantially under-estimated the ability of pre-admission variables such as UGPA and GRE scores to predict subsequent achievement. Therefore, we echo others' calls for further studies and meta-analyses that investigate the use of the GRE and similar standardized tests (11, 28, 39) when accounting for range restriction. Notwithstanding potential weaknesses in such examinations, studies such as the present study highlight their practical utility. Second, assumptions regarding correlations among pre-admissions variables and post-admissions variables should be re-explored with range restriction in mind. In the present study, the average Adj. *R*^2^ for the regression models using UGPA and GRE Verbal to explain variance in VEA, VGPA and NAVLE were 0.71, 0.77, and 0.73, respectively. These findings do not suggest significantly stronger relations between preadmission variables and earlier measures of achievement in veterinary school (e.g., VEA) than later measures of achievement (e.g., NAVLE). Therefore, it is possible that relations between all of these variables have more to do with students' stable overall ability, or capacity to “test well” than any change in their achievement related to veterinary medicine.

Finally, because women constitute the overwhelming majority of participants in the study, and because the number of underrepresented individuals was negligibly small, the present study did not clarify questions regarding gender or race specific biases in the GRE. However, it is clear that many women were admitted notwithstanding the GRE requirement, and that the GRE effectively predicted subsequent achievement for women. Similarly, while students from racial groups historically underrepresented in veterinary medicine were relatively few in this study, they were represented in all cohorts, and therefore constituted part of the sample for which GRE Verbal sub scores contributed significantly to predicting achievement. Therefore, programs with concerns regarding how GRE Verbal scores might impact admissions for women or racially underrepresented groups may consider mechanisms for mitigating potential subgroup differences other than eliminating the GRE Verbal all together from their admissions requirements.

### Limitations

There are several limitations to the present study. First, because it represents only one veterinary program, findings may not generalize to other veterinary programs, or to programs in other fields of study. Second, there are important dependent variables that may not be adequately measured by VEA, NAVLE scores, or overall CVM GPA, such as hands-on clinical skill, communication and professionalism. We join the call for additional research that explores the relation between pre-admissions variables and a broad array of relevant indicators of achievement. Finally, we relied on the best available data when correcting for restriction of range. However, because the available data for the unrestricted sample did not overlap at all (UGPA) or only overlapped partially (GRE Verbal) with our dataset, it is possible that some error was present when calculating range restriction, and that corrected correlations would have been different, if data that exactly matched our cohorts had been available. Nonetheless, the fact that the corrected correlations found in the present study were very similar to those reported by Powers suggests that the corrected correlations are similar to what would have been found had exactly matching data been available.

### Summary

The present study highlights several key implications for admissions decisions, as well as for future research. First, while both undergraduate GPA and GRE scores have been shown to significantly predict subsequent achievement in veterinary colleges, it seems likely that their predictive power has been consistently underestimated because range restriction often has not been taken into account. Veterinary programs that have not considered range restriction for these predictors in their own admissions models may be limiting their ability to predict which applicants will succeed in their programs. Second additional research is needed to establish whether this pattern holds true for other veterinary colleges in other contexts. Finally, in the present study undergraduate GPA and GRE Verbal scores were able to explain a remarkable amount of the variance in overall veterinary GPA, VEA scores, and NAVLE scores. Nonetheless, there continues to be a paucity of evidence regarding how to reliability measure or predict achievement in veterinary clinical practice. Continued research is necessary to address this deficiency and bolster our ability to select and train future veterinarians who will succeed and thrive in their workplaces.

## Data Availability Statement

The datasets presented in this article are not readily available because our research protocol does not permit raw data to be shared. Requests to access the datasets should be directed to jadaniel@iastate.edu.

## Ethics Statement

The studies involving human participants were reviewed and approved by Institutional Review Board - Iowa State University. Written informed consent for participation was not required for this study in accordance with the national legislation and the institutional requirements.

## Author Contributions

JD and RB contributed to planning and conceptualizing the manuscript. RB conducted the data analysis. Both authors contributed to writing and reviewing the manuscript, with RB leading the methods/results sections, and JD leading the other sections.

## Conflict of Interest

The authors declare that the research was conducted in the absence of any commercial or financial relationships that could be construed as a potential conflict of interest.
